# 4-(9-Anthr­yl)-1-(3-bromo­phen­yl)spiro­[azetidine-3,9′-xanthen]-2-one

**DOI:** 10.1107/S1600536809037830

**Published:** 2009-09-26

**Authors:** Ísmail Çelik, Mehmet Akkurt, Aliasghar Jarrahpour, Edris Ebrahimi, Orhan Büyükgüngör

**Affiliations:** a Department of Physics, Faculty of Arts and Sciences, Cumhuriyet University, 58140 Sivas, Turkey; b Department of Physics, Faculty of Arts and Sciences, Erciyes University, 38039 Kayseri, Turkey; c Department of Chemistry, College of Sciences, Shiraz University, 71454 Shiraz, Iran; d Department of Physics, Faculty of Arts and Sciences, Ondokuz Mayıs University, 55139 Samsun, Turkey

## Abstract

In the title mol­ecule, C_35_H_22_BrNO_2_, the four-membered ring of the β-lactam unit is nearly planar [maximum deviation = 0.003 (3) Å] and makes dihedral angles of 87.07 (15), 59.80 (16) and 20.81 (19)°, respectively, with the xanthene system, the anthracene system and the bromo-substituted benzene ring. The mol­ecular conformation is stabilized by weak intra­molecular C—H⋯O and C—H⋯N hydrogen bonds. The crystal structure features weak C—H⋯π inter­actions.

## Related literature

For general background to *β*-lactam anti­biotics, see: Jarrahpour & Khalili (2007 ▶); Landis-Piwowar *et al.* (2006 ▶); Palomo *et al.* (2003 ▶); Skiles & McNeil (1990 ▶); Wu & Tormos (1997 ▶). For related structures, see: Akkurt *et al.* (2006 ▶, 2007 ▶); Akkurt, Jarrahpour *et al.* (2008 ▶); Akkurt, Karaca *et al.* (2008 ▶); Pınar *et al.* (2006 ▶). For geometric analysis, see: Cremer & Pople (1975 ▶).
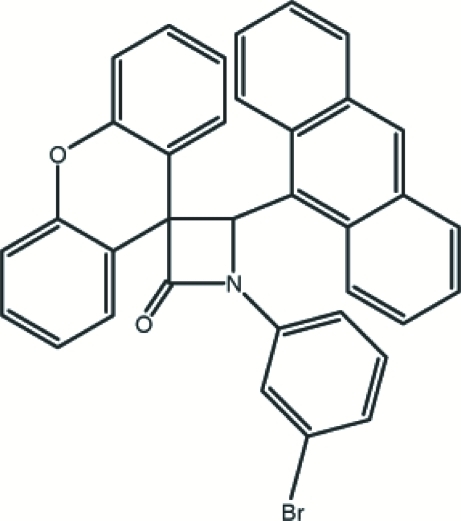


## Experimental

### 

#### Crystal data

C_35_H_22_BrNO_2_
*M* *_r_* = 568.44
Monoclinic, 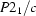

*a* = 11.1143 (4) Å
*b* = 19.9412 (5) Å
*c* = 14.0317 (5) Å
β = 122.106 (2)°
*V* = 2634.28 (16) Å^3^
*Z* = 4
Mo *K*α radiation
μ = 1.59 mm^−1^
*T* = 295 K
0.71 × 0.59 × 0.39 mm

#### Data collection

Stoe IPDS2 diffractometer
Absorption correction: integration (**X-RED32**; Stoe & Cie, 2002 ▶) *T*_min_ = 0.397, *T*_max_ = 0.575
39287 measured reflections
5424 independent reflections
4308 reflections with *I* > 2σ(*I*)
*R*_int_ = 0.046

#### Refinement

*R*[*F* ^2^ > 2σ(*F* ^2^)] = 0.053
*wR*(*F* ^2^) = 0.122
*S* = 1.08
5424 reflections
352 parameters
H-atom parameters constrained
Δρ_max_ = 0.52 e Å^−3^
Δρ_min_ = −0.74 e Å^−3^

### 

Data collection: *X-AREA* (Stoe & Cie, 2002 ▶); cell refinement: *X-AREA*; data reduction: *X-RED32* (Stoe & Cie, 2002 ▶); program(s) used to solve structure: *SIR97* (Altomare *et al.*, 1999 ▶); program(s) used to refine structure: *SHELXL97* (Sheldrick, 2008 ▶); molecular graphics: *ORTEP-3* (Farrugia, 1997 ▶); software used to prepare material for publication: *WinGX* (Farrugia, 1999 ▶).

## Supplementary Material

Crystal structure: contains datablocks global, I. DOI: 10.1107/S1600536809037830/is2462sup1.cif

Structure factors: contains datablocks I. DOI: 10.1107/S1600536809037830/is2462Isup2.hkl

Additional supplementary materials:  crystallographic information; 3D view; checkCIF report

## Figures and Tables

**Table 1:** Hydrogen-bond geometry (Å, °)

*D*—H⋯*A*	*D*—H	H⋯*A*	*D*⋯*A*	*D*—H⋯*A*
C2—H2⋯N1	0.93	2.30	2.968 (4)	128
C31—H31⋯O2	0.93	2.46	3.073 (4)	123
C11—H11⋯*Cg*2^i^	0.93	2.75	3.653 (5)	164
C26—H26⋯*Cg*1^i^	0.93	2.96	3.616 (4)	129

Symmetry code: (i) 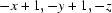. *Cg*1 and *Cg*2 are the centroids of the C8–C13 C30–C35 rings, respectively].

**Fig. 1.**
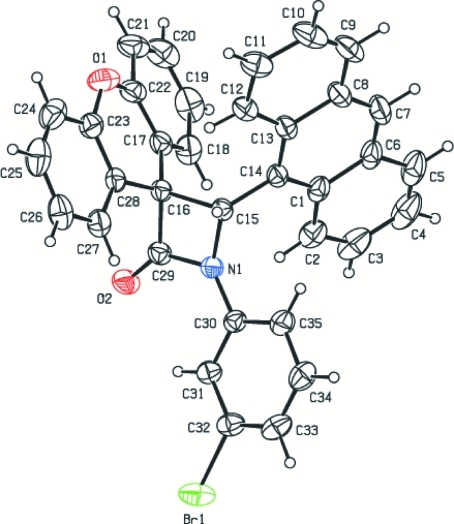
The title molecular structure, with the atom-numbering scheme and 30% probability displacement ellipsoids

**Fig. 2.**
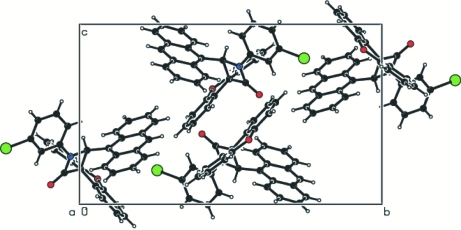
A view down the a axis of the packing of (I).

## supplementary crystallographic information

### Comment

The application of spiro-*β*-lactams in peptidomimetic chemistry is well documented, and relevant examples include the development of constrained *β*-turn mimetics (Palomo *et al.*, 2003). Also, spiro-*β*-lactams have received attention in medicinal chemistry owing to their antiviral and antibacterial properties, (Skiles *et al.*, 1990) as well as recognized activity as cholesterol absorption inhibitors (Wu & Tormos, 1997). Syntheses of new spiro-*β*-lactams have been reported in the literature (Jarrahpour & Khalili, 2007). Persistent but relatively limited research has been devoted to the use of compounds related to polycyclic aromatic hydrocarbons (PAH) asanticancer agents. Banik and co-workers have described the cytotoxicity of a number of new and novel PAH against human cancer cell lines (Landis-Piwowar *et al.*, 2006).

In the title compound (I) (Fig. 1), the *β*-lactam ring (N1/C15/C16/C29) is essentially planar with a maximum deviation of 0.003 (3) Å for C29 from the mean plane and its bond lengths and angles are comparable with the values in our previously papers (Akkurt, Jarrahpour *et al.*, 2008; Akkurt, Karaca *et al.*, 2008; Akkurt *et al.*, 2006,2007; Pınar *et al.*, 2006). Atom O2 lies almost in the *β*-lactam plane, with a deviation of -0.032 (2) Å. The dihedral angle between the benzene ring (C30—C35) attached at N1 and the *β*-lactam ring is 20.81 (19)°.

In the xanthene ring system (O1/C17–C28), attached at C16, the benzene rings (C17–C22) and (C23–C28) are almost planar, forming a dihedral angle of 12.84 (16)° with each other. Its central ring, O1/C16/C17/C22/C23/C28, is not planar, with puckering parameters: Q_T_= 0.198 (3) Å, θ = 99.3 (9)° and φ = 6.5 (9)° (Cremer & Pople, 1975). The mean plane of the xanthene ring system forms the dihedral angles of 87.07 (15)°, and 84.80 (13)°, with the *β*-lactam ring and the benzene ring (C30–C35), respectively.

The anthracene ring system, attached at C15, is almost planar, with maximum deviations of -0.034 (3) Å for C14, 0.032 (3) Å for C13 and 0.031 (4) Å for C1, makes dihedral angle of 59.80 (16)°, 78.58 (13)° and 62.40 (8)°, with the *β*-lactam, benzene and the mean plane of the xanthene ring system, respectively.

Molecular conformation is stabilized by weak intramolecular C—H···O and C—H···N hydrogen bonds. The crystal packing is stabilized by two weak C—H···π interactions [Table 1; *Cg*1 and *Cg*2 refer to the ring centroids of the rings (C8–C13) and (C30–C35), respectively]. Fig. 2 shows a view down the *a* axis of the crystal packing of compound (I).

### Experimental

A mixture of (*E*)-*N*-(anthracen-10-ylmethylene)-3-bromobenzenamine (0.30 g, 0.83 mmol) and triethylamine (0.42 g, 4.15 mmol), 9*H*-xanthen-9-carboxylic acid (0.28 g, 1.24 mmol) and tosyl chloride (0.24 g, 1.24 mmol) in CH_2_Cl_2_(15 ml) was stirred at room temperature for 24 h. Then it was washed with HCl 1 N (20 ml) and saturated sodiumbicarbonate solution (20 ml), brine (20 ml), dried (Na_2_SO_4_) and the solvent was evaporated to give the crude product as an orange crystal which was then purified by recrystallization from ethyl acetate (yield: 55%, m.p.: 495–497 K). IR (KBr, cm^-1^): 1755 (CO β-lactam). ^1^H-NMR δ (p.p.m.): 6.18 (s, 1H, H-4), 6.23–8.65 (m, ArH, 21H).^13^C-NMR δ (p.p.m.): 66.0 (C-3), 75.6 (C-4), 115.7–151.9 (aromatic carbon), 167.7 (CO *β*-lactam). Analysis calculated for C_35_H_22_BrNO_2_: C 73.95, H 3.90, N 2.46%. Found: C 73.90, H 3.93, N 2.51%.

### Refinement

H atoms were positioned geometrically and refined a riding model, with the C—H = 0.93 and 0.98 Å and with *U*_iso_(H) = 1.2*U*_eq_(C).

### Crystal data

**Table:**

C_35_H_22_BrNO_2_	*F*(000) = 1160
*M**_r_* = 568.44	*D*_x_ = 1.433 Mg m^−^^3^
Monoclinic, *P*2_1_/*c*	Mo *K*α radiation, λ = 0.71073 Å
Hall symbol: -P 2ybc	Cell parameters from 43756 reflections
*a* = 11.1143 (4) Å	θ = 1.7–28.0°
*b* = 19.9412 (5) Å	µ = 1.59 mm^−^^1^
*c* = 14.0317 (5) Å	*T* = 295 K
β = 122.106 (2)°	Block, light yellow
*V* = 2634.28 (16) Å^3^	0.71 × 0.59 × 0.39 mm
*Z* = 4	

### Data collection

**Table:**

Stoe IPDS2 diffractometer	5424 independent reflections
Radiation source: sealed X-ray tube, 12 x 0.4 mm long-fine focus	4308 reflections with *I* > 2σ(*I*)
plane graphite	*R*_int_ = 0.046
Detector resolution: 6.67 pixels mm^-1^	θ_max_ = 26.5°, θ_min_ = 2.0°
ω scans	*h* = −13→13
Absorption correction: integration (*X-RED32*; Stoe & Cie, 2002)	*k* = −24→24
*T*_min_ = 0.397, *T*_max_ = 0.575	*l* = −17→17
39287 measured reflections	

### Refinement

**Table:**

Refinement on *F*^2^	Primary atom site location: structure-invariant direct methods
Least-squares matrix: full	Secondary atom site location: difference Fourier map
*R*[*F*^2^ > 2σ(*F*^2^)] = 0.053	Hydrogen site location: inferred from neighbouring sites
*wR*(*F*^2^) = 0.122	H-atom parameters constrained
*S* = 1.08	*w* = 1/[σ^2^(*F*_o_^2^) + (0.0466*P*)^2^ + 1.4948*P*] where *P* = (*F*_o_^2^ + 2*F*_c_^2^)/3
5424 reflections	(Δ/σ)_max_ < 0.001
352 parameters	Δρ_max_ = 0.52 e Å^−^^3^
0 restraints	Δρ_min_ = −0.74 e Å^−^^3^

### Special details

**Table:**

Geometry. Bond distances, angles *etc*. have been calculated using the rounded fractional coordinates. All su's are estimated from the variances of the (full) variance-covariance matrix. The cell e.s.d.'s are taken into account in the estimation of distances, angles and torsion angles
Refinement. Refinement on *F*^2^ for ALL reflections except those flagged by the user for potential systematic errors. Weighted *R*-factors *wR* and all goodnesses of fit *S* are based on *F*^2^, conventional *R*-factors *R* are based on *F*, with *F* set to zero for negative *F*^2^. The observed criterion of *F*^2^ > σ(*F*^2^) is used only for calculating -*R*-factor-obs *etc*. and is not relevant to the choice of reflections for refinement. *R*-factors based on *F*^2^ are statistically about twice as large as those based on *F*, and *R*-factors based on ALL data will be even larger.

### Fractional atomic coordinates and isotropic or equivalent isotropic displacement parameters (Å^2^)

**Table:**

	*x*	*y*	*z*	*U*_iso_*/*U*_eq_	
Br1	0.14849 (5)	0.25728 (2)	0.18916 (4)	0.0883 (2)	
O1	0.9308 (2)	0.56016 (12)	0.3586 (2)	0.0757 (8)	
O2	0.6106 (2)	0.40529 (10)	0.39082 (18)	0.0675 (7)	
N1	0.4500 (2)	0.47038 (10)	0.23373 (18)	0.0475 (7)	
C1	0.3676 (3)	0.62105 (14)	0.2257 (2)	0.0543 (8)	
C2	0.3254 (3)	0.58361 (19)	0.2902 (3)	0.0675 (11)	
C3	0.2423 (4)	0.6108 (2)	0.3248 (3)	0.0878 (14)	
C4	0.1913 (4)	0.6773 (3)	0.2953 (4)	0.1023 (18)	
C5	0.2282 (4)	0.7145 (2)	0.2365 (4)	0.0895 (16)	
C6	0.3177 (3)	0.68965 (16)	0.1994 (3)	0.0668 (10)	
C7	0.3553 (4)	0.72966 (16)	0.1396 (3)	0.0732 (11)	
C8	0.4436 (3)	0.70807 (14)	0.1049 (2)	0.0617 (9)	
C9	0.4806 (4)	0.74926 (15)	0.0411 (3)	0.0798 (13)	
C10	0.5646 (5)	0.72759 (18)	0.0068 (3)	0.0841 (14)	
C11	0.6241 (4)	0.66303 (17)	0.0374 (3)	0.0724 (12)	
C12	0.5919 (3)	0.62105 (14)	0.0967 (2)	0.0562 (9)	
C13	0.4974 (3)	0.64013 (13)	0.1315 (2)	0.0500 (8)	
C14	0.4549 (3)	0.59686 (12)	0.1883 (2)	0.0461 (8)	
C15	0.5078 (3)	0.52549 (12)	0.1999 (2)	0.0429 (7)	
C16	0.6615 (3)	0.50414 (12)	0.3045 (2)	0.0451 (7)	
C17	0.7443 (3)	0.55389 (13)	0.3976 (2)	0.0516 (8)	
C18	0.6972 (4)	0.57626 (17)	0.4658 (3)	0.0684 (11)	
C19	0.7729 (5)	0.62392 (19)	0.5495 (3)	0.0836 (14)	
C20	0.8975 (5)	0.64880 (19)	0.5662 (3)	0.0892 (14)	
C21	0.9491 (4)	0.62630 (17)	0.5034 (3)	0.0784 (11)	
C22	0.8721 (3)	0.57905 (14)	0.4188 (2)	0.0595 (9)	
C23	0.8774 (3)	0.50458 (15)	0.2907 (2)	0.0584 (10)	
C24	0.9551 (4)	0.4803 (2)	0.2475 (3)	0.0804 (14)	
C25	0.9094 (4)	0.4251 (2)	0.1796 (3)	0.0851 (16)	
C26	0.7873 (4)	0.39262 (19)	0.1543 (3)	0.0776 (12)	
C27	0.7097 (3)	0.41707 (15)	0.1974 (3)	0.0610 (10)	
C28	0.7521 (3)	0.47399 (13)	0.2655 (2)	0.0485 (8)	
C29	0.5791 (3)	0.45045 (13)	0.3248 (2)	0.0498 (8)	
C30	0.3212 (3)	0.43540 (13)	0.1680 (2)	0.0460 (8)	
C31	0.3017 (3)	0.37439 (13)	0.2052 (2)	0.0500 (8)	
C32	0.1775 (3)	0.33946 (14)	0.1364 (3)	0.0561 (10)	
C33	0.0742 (3)	0.36319 (18)	0.0326 (3)	0.0667 (11)	
C34	0.0942 (3)	0.42442 (19)	−0.0021 (3)	0.0703 (11)	
C35	0.2170 (3)	0.46130 (16)	0.0651 (2)	0.0601 (10)	
H2	0.35540	0.53940	0.30900	0.0810*	
H3	0.21890	0.58540	0.36830	0.1050*	
H4	0.13200	0.69490	0.31710	0.1230*	
H5	0.19490	0.75840	0.21870	0.1070*	
H7	0.31960	0.77310	0.12190	0.0880*	
H9	0.44480	0.79270	0.02290	0.0950*	
H10	0.58390	0.75500	−0.03710	0.1010*	
H11	0.68670	0.64890	0.01650	0.0870*	
H12	0.63270	0.57860	0.11530	0.0670*	
H15	0.50170	0.51280	0.13010	0.0520*	
H18	0.61330	0.55910	0.45540	0.0820*	
H19	0.73900	0.63880	0.59370	0.1000*	
H20	0.94720	0.68130	0.62100	0.1070*	
H21	1.03530	0.64230	0.51670	0.0940*	
H24	1.03880	0.50150	0.26460	0.0960*	
H25	0.96180	0.40930	0.15020	0.1020*	
H26	0.75710	0.35470	0.10880	0.0930*	
H27	0.62690	0.39500	0.18060	0.0730*	
H31	0.37060	0.35740	0.27510	0.0600*	
H33	−0.00770	0.33840	−0.01330	0.0800*	
H34	0.02440	0.44130	−0.07170	0.0850*	
H35	0.22910	0.50290	0.04130	0.0720*	

### Atomic displacement parameters (Å^2^)

**Table:**

	*U*^11^	*U*^22^	*U*^33^	*U*^12^	*U*^13^	*U*^23^
Br1	0.0990 (3)	0.0679 (2)	0.0934 (3)	−0.0370 (2)	0.0481 (2)	−0.0161 (2)
O1	0.0685 (14)	0.0753 (15)	0.0782 (15)	−0.0261 (11)	0.0355 (13)	−0.0033 (12)
O2	0.0671 (13)	0.0592 (12)	0.0570 (12)	−0.0049 (10)	0.0201 (10)	0.0178 (10)
N1	0.0440 (11)	0.0440 (11)	0.0460 (12)	−0.0020 (9)	0.0182 (10)	0.0036 (9)
C1	0.0395 (13)	0.0594 (16)	0.0496 (15)	0.0022 (11)	0.0140 (12)	−0.0148 (13)
C2	0.0560 (17)	0.082 (2)	0.0633 (19)	−0.0060 (15)	0.0310 (16)	−0.0190 (16)
C3	0.062 (2)	0.126 (3)	0.081 (2)	−0.018 (2)	0.0418 (19)	−0.042 (2)
C4	0.058 (2)	0.139 (4)	0.104 (3)	0.001 (2)	0.039 (2)	−0.062 (3)
C5	0.057 (2)	0.095 (3)	0.092 (3)	0.0152 (19)	0.023 (2)	−0.041 (2)
C6	0.0428 (15)	0.0620 (18)	0.067 (2)	0.0086 (13)	0.0099 (14)	−0.0245 (16)
C7	0.066 (2)	0.0451 (16)	0.072 (2)	0.0148 (14)	0.0120 (17)	−0.0073 (15)
C8	0.0629 (17)	0.0399 (14)	0.0537 (17)	0.0027 (12)	0.0116 (14)	−0.0003 (12)
C9	0.095 (3)	0.0402 (16)	0.065 (2)	−0.0026 (15)	0.0160 (19)	0.0091 (14)
C10	0.119 (3)	0.058 (2)	0.065 (2)	−0.017 (2)	0.042 (2)	0.0067 (16)
C11	0.091 (2)	0.065 (2)	0.063 (2)	−0.0138 (17)	0.0422 (19)	−0.0021 (16)
C12	0.0659 (17)	0.0474 (15)	0.0535 (16)	−0.0011 (12)	0.0305 (14)	0.0029 (12)
C13	0.0519 (14)	0.0396 (13)	0.0429 (14)	0.0033 (11)	0.0146 (12)	−0.0009 (11)
C14	0.0417 (13)	0.0425 (13)	0.0392 (13)	0.0026 (10)	0.0114 (11)	−0.0028 (10)
C15	0.0440 (13)	0.0397 (12)	0.0397 (13)	0.0024 (10)	0.0186 (11)	0.0008 (10)
C16	0.0416 (13)	0.0400 (12)	0.0423 (13)	0.0007 (10)	0.0146 (11)	0.0035 (10)
C17	0.0531 (15)	0.0422 (13)	0.0418 (14)	0.0028 (11)	0.0133 (12)	0.0013 (11)
C18	0.0655 (19)	0.070 (2)	0.0535 (17)	0.0061 (15)	0.0206 (15)	−0.0107 (15)
C19	0.100 (3)	0.077 (2)	0.0517 (19)	0.015 (2)	0.0255 (19)	−0.0099 (17)
C20	0.109 (3)	0.064 (2)	0.053 (2)	−0.014 (2)	0.015 (2)	−0.0081 (16)
C21	0.079 (2)	0.0619 (19)	0.0574 (19)	−0.0243 (17)	0.0113 (18)	0.0043 (16)
C22	0.0601 (17)	0.0477 (15)	0.0503 (16)	−0.0085 (13)	0.0155 (14)	0.0069 (12)
C23	0.0534 (16)	0.0610 (17)	0.0547 (17)	0.0040 (13)	0.0247 (14)	0.0127 (13)
C24	0.061 (2)	0.106 (3)	0.078 (2)	0.0084 (19)	0.0396 (19)	0.022 (2)
C25	0.081 (3)	0.113 (3)	0.070 (2)	0.037 (2)	0.046 (2)	0.018 (2)
C26	0.087 (2)	0.077 (2)	0.060 (2)	0.0271 (19)	0.0331 (18)	0.0004 (16)
C27	0.0577 (17)	0.0576 (17)	0.0567 (17)	0.0084 (13)	0.0229 (14)	−0.0045 (13)
C28	0.0445 (13)	0.0453 (13)	0.0477 (14)	0.0081 (11)	0.0191 (12)	0.0074 (11)
C29	0.0524 (15)	0.0448 (14)	0.0446 (14)	−0.0014 (11)	0.0207 (12)	0.0011 (11)
C30	0.0424 (13)	0.0476 (14)	0.0470 (14)	−0.0026 (10)	0.0231 (12)	−0.0064 (11)
C31	0.0509 (14)	0.0525 (15)	0.0471 (15)	−0.0047 (11)	0.0263 (12)	−0.0054 (12)
C32	0.0595 (17)	0.0579 (16)	0.0585 (18)	−0.0134 (13)	0.0364 (15)	−0.0165 (13)
C33	0.0526 (17)	0.087 (2)	0.0598 (19)	−0.0195 (16)	0.0294 (15)	−0.0208 (17)
C34	0.0510 (17)	0.091 (2)	0.0540 (18)	−0.0013 (16)	0.0179 (15)	0.0008 (17)
C35	0.0481 (15)	0.0654 (18)	0.0539 (17)	−0.0017 (13)	0.0184 (13)	0.0013 (14)

### Geometric parameters (Å, °)

**Table:**

Br1—C32	1.895 (3)	C23—C24	1.380 (6)
O1—C22	1.366 (4)	C23—C28	1.383 (5)
O1—C23	1.374 (4)	C24—C25	1.365 (5)
O2—C29	1.203 (3)	C25—C26	1.369 (7)
N1—C15	1.473 (4)	C26—C27	1.379 (6)
N1—C29	1.379 (4)	C27—C28	1.395 (4)
N1—C30	1.408 (4)	C30—C31	1.386 (4)
C1—C2	1.431 (5)	C30—C35	1.384 (4)
C1—C6	1.448 (4)	C31—C32	1.381 (5)
C1—C14	1.411 (5)	C32—C33	1.373 (5)
C2—C3	1.364 (6)	C33—C34	1.376 (5)
C3—C4	1.415 (7)	C34—C35	1.387 (5)
C4—C5	1.326 (7)	C2—H2	0.9300
C5—C6	1.434 (6)	C3—H3	0.9300
C6—C7	1.374 (5)	C4—H4	0.9300
C7—C8	1.376 (6)	C5—H5	0.9300
C8—C9	1.427 (5)	C7—H7	0.9300
C8—C13	1.448 (4)	C9—H9	0.9300
C9—C10	1.330 (7)	C10—H10	0.9300
C10—C11	1.406 (5)	C11—H11	0.9300
C11—C12	1.356 (5)	C12—H12	0.9300
C12—C13	1.425 (5)	C15—H15	0.9800
C13—C14	1.415 (4)	C18—H18	0.9300
C14—C15	1.515 (4)	C19—H19	0.9300
C15—C16	1.609 (4)	C20—H20	0.9300
C16—C17	1.503 (3)	C21—H21	0.9300
C16—C28	1.504 (5)	C24—H24	0.9300
C16—C29	1.531 (4)	C25—H25	0.9300
C17—C18	1.386 (5)	C26—H26	0.9300
C17—C22	1.382 (5)	C27—H27	0.9300
C18—C19	1.393 (5)	C31—H31	0.9300
C19—C20	1.369 (8)	C33—H33	0.9300
C20—C21	1.358 (7)	C34—H34	0.9300
C21—C22	1.396 (4)	C35—H35	0.9300
			
Br1···C21^i^	3.491 (4)	C29···H2	2.9600
O2···C31	3.073 (4)	C30···H2	2.7500
O2···H31	2.4600	C30···H11^ix^	3.0500
O2···H7^i^	2.7800	C31···H19^ii^	3.1000
O2···H9^i^	2.7700	C32···H25^vii^	2.8700
O2···H3^ii^	2.8700	C33···H11^ix^	3.0900
N1···C2	2.968 (4)	C33···H25^vii^	2.7100
N1···C27	3.359 (5)	C34···H11^ix^	2.9400
N1···H2	2.3000	C35···H15	2.9700
N1···H27	2.8700	C35···H11^ix^	2.9300
C1···C18	3.528 (5)	H2···N1	2.3000
C2···N1	2.968 (4)	H2···C15	2.8400
C2···C18	3.514 (6)	H2···C29	2.9600
C2···C30	3.405 (5)	H2···C30	2.7500
C4···C9^iii^	3.548 (6)	H2···H18	2.5200
C9···C4^iv^	3.548 (6)	H3···H24^vii^	2.4100
C12···C16	3.484 (4)	H3···O2^ii^	2.8700
C12···C28	3.591 (4)	H5···H7	2.4200
C14···C35	3.525 (4)	H7···H5	2.4200
C14···C18	3.382 (4)	H7···H9	2.4600
C16···C12	3.484 (4)	H7···O2^vi^	2.7800
C18···C14	3.382 (4)	H9···H7	2.4600
C18···C2	3.514 (6)	H9···O2^vi^	2.7700
C18···C1	3.528 (5)	H9···C4^iv^	2.9900
C19···C24^v^	3.524 (6)	H10···C19^iv^	3.0000
C20···C25^v^	3.371 (5)	H11···C30^ix^	3.0500
C20···C24^v^	3.405 (5)	H11···C33^ix^	3.0900
C21···C23^v^	3.599 (4)	H11···C34^ix^	2.9400
C21···Br1^vi^	3.491 (4)	H11···C35^ix^	2.9300
C23···C21^v^	3.599 (4)	H12···C15	2.4900
C24···C20^v^	3.405 (5)	H12···C16	2.9100
C24···C19^v^	3.524 (6)	H12···C23	2.9200
C25···C20^v^	3.371 (5)	H12···C28	2.7500
C27···N1	3.359 (5)	H12···H15	2.0500
C28···C12	3.591 (4)	H15···C12	2.5300
C30···C2	3.405 (5)	H15···C27	2.7500
C31···O2	3.073 (4)	H15···C35	2.9700
C35···C14	3.525 (4)	H15···H12	2.0500
C2···H18	2.8200	H15···H35	2.6000
C3···H24^vii^	2.9200	H18···C2	2.8200
C4···H9^iii^	2.9900	H18···C29	2.7300
C5···H33^viii^	2.9600	H18···H2	2.5200
C6···H33^viii^	3.0700	H19···C31^ii^	3.1000
C8···H26^ix^	2.9000	H24···C3^x^	2.9200
C9···H31^vi^	3.0800	H24···H3^x^	2.4100
C11···H27^ix^	3.0700	H25···C32^x^	2.8700
C12···H15	2.5300	H25···C33^x^	2.7100
C13···H26^ix^	3.0400	H26···C8^ix^	2.9000
C14···H35	2.9300	H26···C13^ix^	3.0400
C15···H12	2.4900	H27···N1	2.8700
C15···H27	3.0000	H27···C15	3.0000
C15···H2	2.8400	H27···C29	2.6000
C15···H35	2.7200	H27···C11^ix^	3.0700
C16···H12	2.9100	H31···O2	2.4600
C19···H10^iii^	3.0000	H31···C29	2.7500
C23···H12	2.9200	H31···C9^i^	3.0800
C24···H34^ix^	3.0300	H33···C5^viii^	2.9600
C25···H35^ix^	2.9900	H33···C6^viii^	3.0700
C27···H15	2.7500	H34···C24^ix^	3.0300
C28···H12	2.7500	H35···C14	2.9300
C29···H27	2.6000	H35···C15	2.7200
C29···H18	2.7300	H35···H15	2.6000
C29···H31	2.7500	H35···C25^ix^	2.9900
			
C22—O1—C23	118.6 (3)	O2—C29—C16	135.1 (3)
C15—N1—C29	95.5 (2)	N1—C29—C16	93.3 (2)
C15—N1—C30	128.6 (2)	N1—C30—C31	119.8 (2)
C29—N1—C30	131.2 (2)	N1—C30—C35	119.6 (3)
C2—C1—C6	116.4 (3)	C31—C30—C35	120.6 (3)
C2—C1—C14	125.3 (3)	C30—C31—C32	118.6 (3)
C6—C1—C14	118.3 (3)	Br1—C32—C31	118.5 (2)
C1—C2—C3	122.0 (3)	Br1—C32—C33	119.5 (3)
C2—C3—C4	120.7 (4)	C31—C32—C33	122.0 (3)
C3—C4—C5	119.9 (5)	C32—C33—C34	118.7 (3)
C4—C5—C6	122.4 (4)	C33—C34—C35	121.0 (3)
C1—C6—C5	118.7 (3)	C30—C35—C34	119.1 (3)
C1—C6—C7	120.3 (3)	C1—C2—H2	119.00
C5—C6—C7	121.1 (3)	C3—C2—H2	119.00
C6—C7—C8	122.7 (3)	C2—C3—H3	120.00
C7—C8—C9	122.6 (3)	C4—C3—H3	120.00
C7—C8—C13	118.4 (3)	C3—C4—H4	120.00
C9—C8—C13	118.9 (3)	C5—C4—H4	120.00
C8—C9—C10	122.1 (3)	C4—C5—H5	119.00
C9—C10—C11	119.6 (4)	C6—C5—H5	119.00
C10—C11—C12	121.2 (4)	C6—C7—H7	119.00
C11—C12—C13	121.9 (3)	C8—C7—H7	119.00
C8—C13—C12	116.1 (3)	C8—C9—H9	119.00
C8—C13—C14	120.0 (3)	C10—C9—H9	119.00
C12—C13—C14	123.9 (3)	C9—C10—H10	120.00
C1—C14—C13	120.2 (2)	C11—C10—H10	120.00
C1—C14—C15	125.8 (3)	C10—C11—H11	119.00
C13—C14—C15	114.0 (3)	C12—C11—H11	119.00
N1—C15—C14	121.6 (3)	C11—C12—H12	119.00
N1—C15—C16	86.68 (19)	C13—C12—H12	119.00
C14—C15—C16	120.7 (2)	N1—C15—H15	109.00
C15—C16—C17	118.7 (2)	C14—C15—H15	109.00
C15—C16—C28	111.4 (2)	C16—C15—H15	109.00
C15—C16—C29	84.5 (2)	C17—C18—H18	119.00
C17—C16—C28	111.2 (3)	C19—C18—H18	119.00
C17—C16—C29	116.8 (2)	C18—C19—H19	120.00
C28—C16—C29	111.8 (2)	C20—C19—H19	120.00
C16—C17—C18	122.1 (3)	C19—C20—H20	120.00
C16—C17—C22	120.8 (3)	C21—C20—H20	120.00
C18—C17—C22	117.2 (3)	C20—C21—H21	120.00
C17—C18—C19	121.4 (4)	C22—C21—H21	120.00
C18—C19—C20	119.6 (5)	C23—C24—H24	120.00
C19—C20—C21	120.5 (4)	C25—C24—H24	120.00
C20—C21—C22	119.7 (4)	C24—C25—H25	120.00
O1—C22—C17	122.7 (2)	C26—C25—H25	120.00
O1—C22—C21	115.7 (3)	C25—C26—H26	121.00
C17—C22—C21	121.6 (3)	C27—C26—H26	120.00
O1—C23—C24	116.6 (3)	C26—C27—H27	119.00
O1—C23—C28	122.6 (3)	C28—C27—H27	119.00
C24—C23—C28	120.9 (3)	C30—C31—H31	121.00
C23—C24—C25	120.1 (4)	C32—C31—H31	121.00
C24—C25—C26	120.8 (4)	C32—C33—H33	121.00
C25—C26—C27	118.9 (3)	C34—C33—H33	121.00
C26—C27—C28	121.7 (4)	C33—C34—H34	119.00
C16—C28—C23	120.6 (2)	C35—C34—H34	120.00
C16—C28—C27	121.8 (3)	C30—C35—H35	120.00
C23—C28—C27	117.5 (3)	C34—C35—H35	120.00
O2—C29—N1	131.5 (3)		
			
C23—O1—C22—C21	166.8 (3)	C14—C15—C16—C17	7.2 (4)
C22—O1—C23—C24	−168.8 (3)	N1—C15—C16—C17	−118.0 (3)
C22—O1—C23—C28	11.5 (4)	N1—C15—C16—C28	110.9 (2)
C23—O1—C22—C17	−13.8 (4)	C14—C15—C16—C29	124.8 (3)
C29—N1—C30—C31	13.3 (5)	C14—C15—C16—C28	−123.9 (3)
C29—N1—C30—C35	−164.7 (3)	C29—C16—C28—C27	33.9 (3)
C29—N1—C15—C16	0.4 (2)	C29—C16—C28—C23	−150.1 (2)
C15—N1—C30—C35	−14.9 (5)	C15—C16—C28—C27	−58.7 (3)
C30—N1—C15—C14	78.4 (3)	C15—C16—C29—O2	177.7 (4)
C15—N1—C29—C16	−0.4 (2)	C15—C16—C28—C23	117.3 (3)
C30—N1—C15—C16	−157.3 (3)	C28—C16—C17—C18	−164.1 (3)
C29—N1—C15—C14	−123.9 (2)	C28—C16—C17—C22	15.6 (3)
C30—N1—C29—C16	156.3 (3)	C29—C16—C17—C18	−34.3 (4)
C15—N1—C30—C31	163.1 (3)	C29—C16—C17—C22	145.5 (3)
C15—N1—C29—O2	−177.8 (3)	C15—C16—C17—C18	64.6 (4)
C30—N1—C29—O2	−21.1 (5)	C17—C16—C28—C23	−17.7 (3)
C2—C1—C14—C13	−175.7 (3)	C17—C16—C28—C27	166.4 (3)
C2—C1—C6—C7	178.8 (3)	C15—C16—C29—N1	0.4 (2)
C6—C1—C14—C15	−175.1 (3)	C15—C16—C17—C22	−115.6 (3)
C14—C1—C6—C5	179.6 (3)	C28—C16—C29—N1	−110.5 (2)
C2—C1—C6—C5	−1.5 (5)	C28—C16—C29—O2	66.7 (4)
C14—C1—C6—C7	−0.1 (5)	C17—C16—C29—O2	−62.8 (5)
C14—C1—C2—C3	179.0 (3)	C17—C16—C29—N1	119.9 (3)
C6—C1—C14—C13	3.1 (4)	C16—C17—C22—C21	178.7 (3)
C6—C1—C2—C3	0.2 (5)	C22—C17—C18—C19	2.3 (5)
C2—C1—C14—C15	6.2 (5)	C18—C17—C22—O1	179.0 (3)
C1—C2—C3—C4	1.6 (6)	C16—C17—C18—C19	−178.0 (3)
C2—C3—C4—C5	−2.2 (7)	C18—C17—C22—C21	−1.6 (4)
C3—C4—C5—C6	0.9 (7)	C16—C17—C22—O1	−0.7 (4)
C4—C5—C6—C1	1.0 (6)	C17—C18—C19—C20	−0.8 (5)
C4—C5—C6—C7	−179.3 (4)	C18—C19—C20—C21	−1.4 (6)
C1—C6—C7—C8	−1.9 (5)	C19—C20—C21—C22	2.1 (5)
C5—C6—C7—C8	178.4 (4)	C20—C21—C22—C17	−0.6 (5)
C6—C7—C8—C13	0.8 (5)	C20—C21—C22—O1	178.9 (3)
C6—C7—C8—C9	178.9 (3)	O1—C23—C24—C25	179.8 (3)
C7—C8—C13—C14	2.3 (4)	C28—C23—C24—C25	−0.5 (5)
C7—C8—C9—C10	−179.1 (4)	O1—C23—C28—C16	5.1 (4)
C13—C8—C9—C10	−1.1 (5)	C24—C23—C28—C16	−174.6 (3)
C9—C8—C13—C12	3.7 (4)	C24—C23—C28—C27	1.5 (4)
C7—C8—C13—C12	−178.1 (3)	O1—C23—C28—C27	−178.8 (3)
C9—C8—C13—C14	−175.9 (3)	C23—C24—C25—C26	−0.7 (6)
C8—C9—C10—C11	−2.5 (6)	C24—C25—C26—C27	0.8 (6)
C9—C10—C11—C12	3.3 (6)	C25—C26—C27—C28	0.3 (5)
C10—C11—C12—C13	−0.4 (5)	C26—C27—C28—C16	174.7 (3)
C11—C12—C13—C8	−3.1 (4)	C26—C27—C28—C23	−1.4 (5)
C11—C12—C13—C14	176.6 (3)	N1—C30—C31—C32	−176.6 (3)
C12—C13—C14—C15	−5.5 (4)	C35—C30—C31—C32	1.3 (5)
C8—C13—C14—C15	174.2 (2)	N1—C30—C35—C34	176.0 (3)
C12—C13—C14—C1	176.2 (3)	C31—C30—C35—C34	−1.9 (5)
C8—C13—C14—C1	−4.2 (4)	C30—C31—C32—Br1	−178.2 (3)
C1—C14—C15—N1	11.7 (4)	C30—C31—C32—C33	0.5 (5)
C1—C14—C15—C16	−94.9 (4)	Br1—C32—C33—C34	177.0 (3)
C13—C14—C15—C16	86.9 (3)	C31—C32—C33—C34	−1.7 (6)
C13—C14—C15—N1	−166.5 (2)	C32—C33—C34—C35	1.1 (6)
N1—C15—C16—C29	−0.37 (19)	C33—C34—C35—C30	0.7 (5)

Symmetry codes: (i) −*x*+1, *y*−1/2, −*z*+1/2; (ii) −*x*+1, −*y*+1, −*z*+1; (iii) *x*, −*y*+3/2, *z*+1/2; (iv) *x*, −*y*+3/2, *z*−1/2; (v) −*x*+2, −*y*+1, −*z*+1; (vi) −*x*+1, *y*+1/2, −*z*+1/2; (vii) *x*−1, *y*, *z*; (viii) −*x*, −*y*+1, −*z*; (ix) −*x*+1, −*y*+1, −*z*; (x) *x*+1, *y*, *z*.

### Hydrogen-bond geometry (Å, °)

**Table:**

*D*—H···*A*	*D*—H	H···*A*	*D*···*A*	*D*—H···*A*
C2—H2···N1	0.93	2.30	2.968 (4)	128
C31—H31···O2	0.93	2.46	3.073 (4)	123
C11—H11···Cg2^ix^	0.93	2.75	3.653 (5)	164
C26—H26···Cg1^ix^	0.93	2.96	3.616 (4)	129

Symmetry codes: (ix) −*x*+1, −*y*+1, −*z*.
